# Prediction of Compressive Strength of Sustainable Foam Concrete Using Individual and Ensemble Machine Learning Approaches

**DOI:** 10.3390/ma15093166

**Published:** 2022-04-27

**Authors:** Haji Sami Ullah, Rao Arsalan Khushnood, Furqan Farooq, Junaid Ahmad, Nikolai Ivanovich Vatin, Dina Yehia Zakaria Ewais

**Affiliations:** 1 NUST Institute of Civil Engineering (NICE), School of Civil and Environmental Engineering (SCEE), National University of Sciences and Technology (NUST), Sector H-12, Islamabad 44000, Pakistan; hajisamiullah96@gmail.com (H.S.U.); arsalan.khushnood@nice.nust.edu.pk (R.A.K.); junaid.ahmad@nice.nust.edu.pk (J.A.); 2 Military Engineer Service (MES), Ministry of Defence (MoD), Rawalpindi 43600, Pakistan; 3 Peter the Great St. Petersburg Polytechnic University, 195291 St. Petersburg, Russia; vatin@mail.ru; 4 Structural Engineering, Faculty of Engineering and Technology, Future University in Egypt, New Cairo 11835, Egypt; dina.yehya@fue.edu.eg

**Keywords:** sustainable concrete, foamed concrete, compressive strength, machine learning, artificial intelligence, ensemble learners

## Abstract

The entraining and distribution of air voids in the concrete matrix is a complex process that makes the mechanical properties of lightweight foamed concrete (LFC) highly unpredictable. To study the complex nature of aerated concrete, a reliable and robust prediction model is required, employing different machine learning (ML) techniques. This study aims to predict the compressive strength of LFC by using a support vector machine (SVM) as an individual learner along with bagging, boosting, and random forest (RF) as a modified ensemble learner. For that purpose, a database of 191 data points was collected from published literature, where the mix design ingredients, i.e., cement content, sand content, water to cement ratio, and foam volume, were chosen to predict the compressive strength of LFC. The 10-K fold cross-validation method and different statistical error and regression tools, i.e., mean absolute error (MAE), root means square error (RMSE), and coefficient of determinant (R^2^), were used to evaluate the performance of the developed ML models. The modified ensemble learner (RF) outperforms all models by yielding a strong correlation of R^2^ = 0.96 along with the lowest statistical error values of MAE = 1.84 MPa and RMSE = 2.52 MPa. Overall, the result suggests that the ensemble learners would significantly enhance the performance and robustness of ML models.

## 1. Introduction

The production of normal concrete consumes a large quantity of cement and natural aggregates, which raises concerns about environmental degradation and sustainability. The emission of carbon dioxide (CO_2_) from cement production plants is considered as one of the main sources of greenhouse gas (GHG) production [1]. It is roughly estimated that cement production plants are responsible for 7–8% of CO_2_ emissions into the atmosphere [1,2,3]. As the production of cement is expected to increase, the percentage of CO_2_ emission also rises rapidly [4]. The production of cement process requires raw materials and fuel, and the continuous mining of these materials will lead to loss of topsoil and deforestation [5]. On the other hand, the continuous usage and quarrying of resources greatly disturb the natural habitats of organisms. From the lithosphere, the construction industry is expected to consume 60% of the extracted materials [6]. Thus, there is a need for the production of concrete that will minimize or replace the use of cement and natural aggregates and transform the construction industry towards sustainability and also be helpful to alleviate the above-mentioned issues.

Foamed concrete (FC) is a lightweight material composed of either cement or mortar paste with entrapped air voids. The LFC is used as an insulating material having interesting structural features [7]. The LFC can also be used as a structural element for short- and long-term purposes [8]. By controlling the dosage of foaming agent in LFC, a broad range of densities (400–1850 kg/m^3^) can be obtained for different application purposes, i.e., insulation, structural, filling grade, partition, etc. [9,10]. The compressive strength of LFC decreases rapidly with a decrease in its dry density [11]. The fracture energy of the FC notched beam is relatively high, around 18 to 25 N/m, with compressive strength of 6.4–14 MPa [12]. It has been estimated that the entrained air bubbles can replace up to 50% of the total concrete volume, which results in less consumption of cement and natural aggregates [13]. The entrained air voids exhibit a strong plasticizing effect, thus increasing the workability of foamed concrete [14]. The strength of FC can also be affected by the shape and size of the sample specimen, loading direction, pore formation method, and curing method [15]. The LFC has been identified as a light, economic, durable, and sustainable construction material [16]. The possibility of replacing concrete volume with entrained air bubbles has enhanced the sustainability feature and reduced the consumption of cement and aggregates in concrete production.

For the production and practical application of sustainable LFC, the optimization of the main ingredient of mix design is very important. The mix design will significantly affect the behavior and performance of LFC [17,18,19]. The strength of LFC is dependent on mix design ingredients, i.e., cement and sand content, water to binder ratio, foam volume, and curing method [20,21,22]. All the significant properties of concrete, such as durability, permeability, resistance to abrasion, etc., can be represented in terms of its compressive strength [23]. The durability and safety of the concrete elements are evaluated in terms of concrete compressive strength and is considered as the most important parameter [24]. The presence of entrained air voids in LFC makes it difficult to estimate the concrete strength accurately. Normally, the strength of concrete samples in the laboratory is calculated by casting and crushing concrete samples of standard dimension after the stipulated time of curing [25]. However, this is a hit-and-trial method that requires extensive laboratory work and is uneconomical and time-consuming.

Nowadays, the evolution in the artificial intelligence (AI) and machine learning (ML) techniques has made it possible to predict and estimate the different physical and mechanical properties of concrete [26,27,28,29,30]. The strength of concrete can be forecasted accurately against different parameters by using different ML techniques, such as classification, regression, and clustering [31,32,33]. The ML technique provides accurate and reliable results as compared to previous regression methods [34]. Different ML techniques, such as random forest (RF), decision tree (DT), deep learning (DL), gene expression programming (GEP), artificial neural network (ANN), and support vector machine (SVM), use pattern recognition ability to resolve a complex engineering problem. In the case of RF and DT, tree-like structures are used to predict the response. The RF technique randomly chooses the important parameters and DT utilizes the whole database with interested parameters and builds multiple prediction trees. The maximum voters with averaged prediction value give an accurate result. The nonlinear computational approach of ANN can resolve complex engineering problems by developing input and output relations without using any specific equation and can solve complex problems having imprecise or incomplete information. SVM is designed to handle nonlinear regression problems with high generalization ability and provides a globally optimal solution. GEP is an advanced form of genetic algorithm based on Darwinian evolution theory and solves complex engineering problems in the form of non-linear parse tree-like structures called expression trees and provides an explicit numerical expression for the practical application of the developed model. Among all the ML techniques, the DL approach uses a robust design algorithm to resolve complex and rigorous engineering problems, and provides better prediction results. Siddique et al. [35] studied the incorporation of bottom ash in self-compacting concrete by using the ANN approach. Similarly, Dantas et al. [36] utilized the ANN technique to evaluate the strength of recycled concrete made from construction wastes. Chou et al. [37] employed SVM and ANN techniques to estimate the load-bearing capacity of concrete. In the research work of Zhang et al. [38], the RF regression method is used to predict and assess the strength of concrete, and the significant input parameters are also discussed.

The ML approach utilizes the pattern recognition technique by using both a database and statistical analysis. The required information is extracted from a large dataset and establishes different relations to simplify the complex pattern and provide a simple resolution. In the ML approach, there are two types of techniques used for prediction modeling. The first is the standard technique, where a single separate ML model is used for prediction. In the second technique, the newly developed ensemble learning algorithms, i.e., bagging, RF, and boosting, are used. Studies suggest that the ensemble learning model results are more adamant and reliable than individual ML models [39]. The individual standard ML approach, i.e., ANN, SVM, GEP, etc., forms the weak learners. In the ensemble learning approach, the training data are used to train several weak learners, which are then integrated into a strong learner. The high-performing ML techniques are used to model the complex concrete nature by incorporating ensemble learning algorithms and classifier generators. The increasing popularity of the ensemble learning approach has been witnessed in the latest prediction modeling studies due to its accuracy in results as compared to individual standard learners [40].

This research aims to evaluate and compare the prediction capability of network and tree-based ML models, i.e., SVM and RF. This study also addressed the enhancement in the performance of models by using ensemble techniques, such as bagging, boosting, and modified ensemble learner (RF). The novelty and significance of the present study are concerned with the prediction and estimation of LFC compressive strength against different combinations of input ingredients, i.e., cement content, sand content, water to cement ratio, and volume of foam, by implementing the ensemble algorithm over individual learners. Different statistical regression and error tools along with the 10-K fold cross-validation approach were used to assess the performance, reliability, and generalization capability of the prediction models.

## 2. Data Collection and Analysis

### 2.1. Development of Data

**Figure 1:**
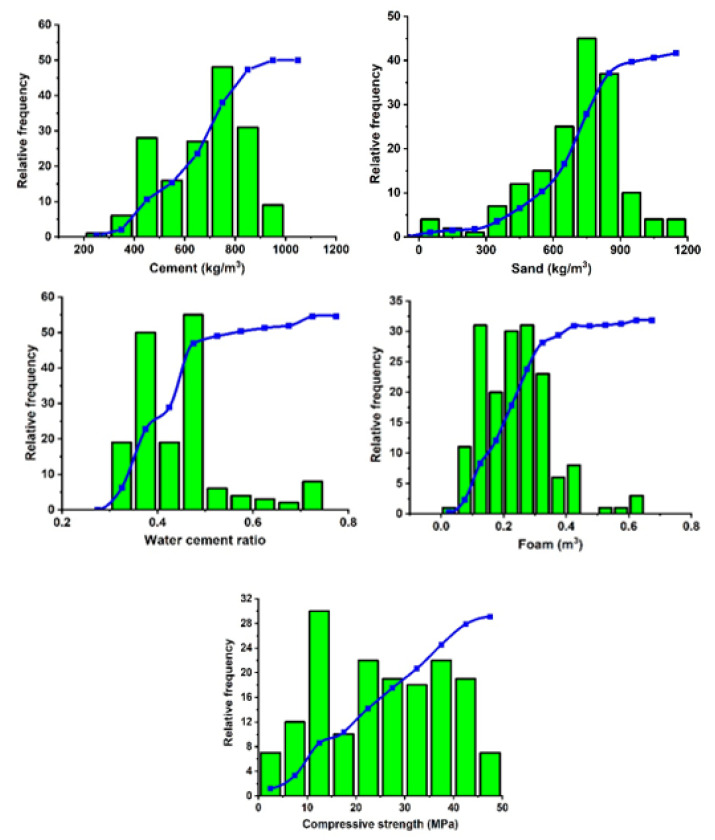
Distribution histogram of collected data.

The required data to develop the ML models was collected from the experimental results of seven different past published literature [2,13,41,42,43,44,45]. The collected database is comprised of 191 data points where the basic mix design ingredients, i.e., cement content (kg/m^3^), sand content (kg/m^3^), water-cement ratio, and foam volume (dm^3^/m^3^) are taken as input, and the 28-day compressive strength of LFC as an output variable. All the compressive strength test results used in this study are cube specimens having the dimensions of (15 × 15 × 15) cm^3^. Table 1 illustrates the statistical description of the collected data, which contain the maximum and minimum ranges, average values, and standard deviation (SD) of all the input and output variables. To obtain a reliable prediction model for the compressive strength of LFC, it is suggested to use the proposed expression within the specified range. The statistical analysis shows that the data covers a large range of mixed design ingredients, and the SD shows the distribution of the data along with its mean value. The greater the SD value, the greater the distribution will be. The distribution histogram of different input variables against the strength of LFC is shown in Figure 1. The histogram shows that the collected data are highly diverse and well distributed. The performance of the AI model is highly dependent on the distribution and dispersion of available data [46]. The collected data of 191 data points were randomly distributed into training and testing data. Here, 80% of data (152 data points) was used to train and develop the ML model, and the other 20% of data (39 data points) was used to evaluate the performance of the prediction model.

### 2.2. Pre-Processing of Data

**Figure 2:**
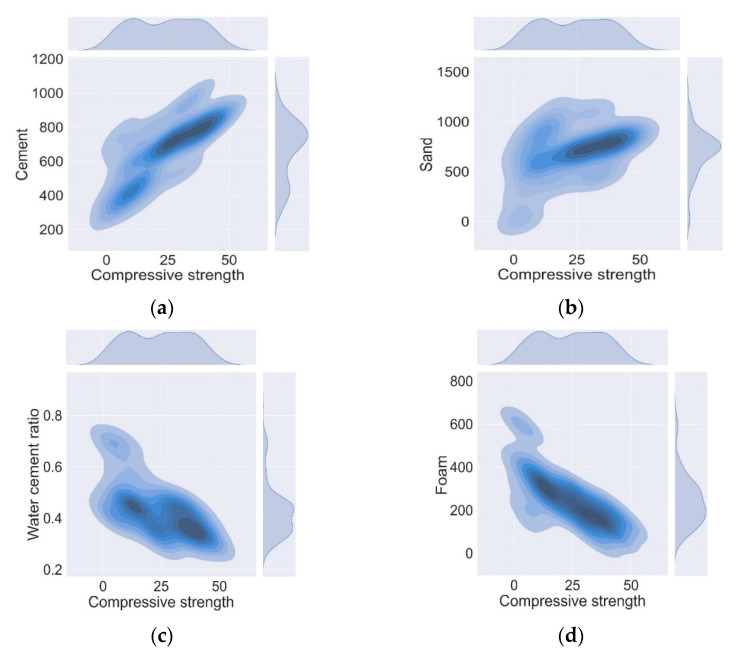
Contour maps of input variables (**a**) cement content; (**b**) sand content; (**c**) w/c ratio; (**d**) foam volume against the compressive strength.

In AI, the pre-processing of data is a key step that is used to evaluate the relation of input and output parameters before the development of any ML models. This step is used to check the validity of the collected data and to assess the trend followed by the output parameter under the influence of the inputs. To avoid any complexity in the assessment of the final ML model, the correlation between the input and output variable is evaluated before the development of the AI model [47]. The Pearson correlation coefficient (r) was evaluated to find out the relation between the given variables [48]. The Pearson correlation (r) matrix of given variables is shown in Table 2 and was calculated by using the statistical software Minitab-16. Here, the ±1 shows a strong correlation and 0 means no relation between the input and output parameters. The positive sign shows a direct relation, and the negative sign means there exists an inverse relationship between the variables. Figure 2 shows the relationship of mix design parameters and the strength of LFC in the form of contour maps, which show that all the input parameters followed the global trend. For example, cement and sand content show a direct relation as shown in Figure 2a,b. Whereas, w/c and foam volume followed the inverse relation as illustrated in Figure 2c,d. The dark colors of contour maps show the intensity of input variables within a range. The results of pre-processing manifest that all the input parameters hold a strong correlation with the compressive strength of LFC and have also followed the global trend. Hence, the collected data are valid and can be used for the development of ML models.

## 3. Methodology

The AI models are developed by training the available data and are calibrated and validated with the laboratory test results. The pattern recognition ability of the AI technique transforms the complex pattern of available data into a simplified pattern to resolve complex engineering problems. Table 3 illustrates the summary of different ML algorithms used in recent years for predicting the various properties of concrete. In this study, the ML approaches are chosen to evaluate and compare the prediction performance of tree and network-based decision-making techniques. The ensemble learning algorithms were applied to individual ML models to further enhance the prediction capability of the developed models. Furthermore, the validity of the models is evaluated by using a 10-K fold cross-validation method and different statistical evaluation tools.

### 3.1. Machine Learning Approach

#### 3.1.1. Random Forest (RF) Regression Models

The RF technique uses both the classification and regression approaches and has been used by different researchers [38,76]. Though DT and RF both work on tree-based decision methods but there is a major difference between them. In DT modeling, a single tree is developed, but the RF technique results in the construction of several trees which are called forests, and the arbitrarily chosen data are assigned to them. The data are provided in matrix form and the different dimensions of rows and columns are selected [77]. Large datasets can be more effectively handled by RF than any other ML technique. There are three main steps in RF regression model development. First, the training dataset is used to assemble the trained regression trees. Then, the mean value is evaluated for single regression tree outcomes, and finally, validation datasets are used to validate the predicted results. The new trained data set, which is comprised of boot-strap data, is calculated from the original data set. The removal and swapping of data points occur and result in the formulation of a new dataset called out-of-bag datapoints, which assembles all the removed data points. In the end, the two by third data points are used for the estimation of the regression function and the developed regression model is validated against the remaining out-of-bag data points. The process continues until the required accuracy is achieved. The deletion of data points in the out-of-bag dataset and using them in validation is a distinctive feature of the RF technique [29]. Finally, the gross error is computed for all expression trees, which manifests the accuracy and effectiveness of each developed tree.

#### 3.1.2. Support Vector Machine (SVM) Models

The SVM is a supervised learner that analyzes the data for classification and regression problems. The SVM approach can generalize and resolve practical problems, such as non-linearity, high input dimensional spaces, and small database problems. To achieve better accuracy, the SVM can transform input space into a high dimensional space with the help of a non-linear transformation, which is defined by an inner product function. The non-linear regression problems are solved efficiently by using SVM regression models [78]. For the classification of data, the regression data are first mapped into the n-dimensional space function. The non-linear kernel functions are used which meet the high dimensional space to enhance the classification and distinction of the original input space data. Equation (1) shows the linear function in space in terms of *f(x,w)*.

(1) $$f\left(x,w\right)=\overset{n}{\underset{j=1}{\sum }}w_{j}g_{j}\left(x\right)+b$$

where *w*, *g_j_(x),* and *b* refer to weight vector transformation, non-linear input space, and bias term respectively. The loss function *Lε* is a measurement of estimation quality and is given in Equation (2).

(2) $$L_{\varepsilon }=L_{\varepsilon }(y,f\left(x,w\right)=\{\begin{array}{c} 0,\;\;if\;\left|y-f\left(x,w\right)\right|\leq \varepsilon \\ \left|y-f\left(x,w\right)\right|,\;\;otherwise \end{array}$$

In the SVM regression approach, the new higher dimensional feature space is computed from the linear regression function by lowering the ${||w||}^{2}$, which also reduces the complexity of model at the same time. The non-negative slack variables $\xi_{i}+\xi_{i}^{*}$ establish the function, where *i* = 1,2, 3…, *n* will identify samples from the π-intensive field. The simplified SVM regression model is constructed from the functions given in Equation (3).

(3) $$min\frac{1}{2}{||w||}^{2}+C\sum_{i=1}^{n}\left(\xi_{i}+\xi_{i}^{*}\right)\;\;\;\;\;subjected\;to\;\{\begin{array}{c} y_{i}-f\left(x_{i},w\right)\leq \varepsilon +\xi_{i}^{*}\\ f\left(x_{i},w\right)-y_{i}\leq \varepsilon +\xi_{i}^{*}\\ \xi_{i},\;\xi_{i}^{*}\geq 0,i=1,\;2,\;\ldots ,\;n \end{array}$$

The optimized problem can be changed into a resolved dual problem and is given in Equation (4).

(4) $$f\left(x\right)=\overset{nsv}{\underset{i=1}{\sum }}(\alpha_{i}+\alpha_{i}^{*})K\left(x,x_{i}\right)\;subject\;to\;0\leq \alpha_{i}^{*}\leq C,\;0\leq \alpha_{i}\leq C$$

where *nsv* = number of support vectors. The kernel function is given in Equation (5).

(5) $$K\left(x,x_{i}\right)=\overset{m}{\underset{i=1}{\sum }}(g_{i}\left(x\right)+g_{i}\left(x_{i}\right))$$

To find the support vector along with the function space, the kernel functions, i.e., linear, polynomial, radial basis, or sigmoid function, are chosen by the training set. It should also be noted that the kernel parameters are also affected by the implemented software and the chosen function.

### 3.2. Ensemble Algorithms Using Bagging and Boosting

The ensemble learners enhance the prediction capability and accuracy of the ML techniques. In ensemble techniques, the training data are combined and aggregated from several weak predictive models to reduce the concern of over-fitting. The formation of an optimal predictive model is achieved from the combination of qualified sub-models (weak predictive models) by using the combining, averaging, and voting approach. In ensemble modeling, bagging is an effective technique that utilizes the bootstrap retesting approach and assembles benefits. In this process, the part models are substituted by the initial training set. There is a possibility that the product models may contain some data points several times and some data points may be ignored. The outputs of component models are averaged to obtain the final output.

**Figure 3:**
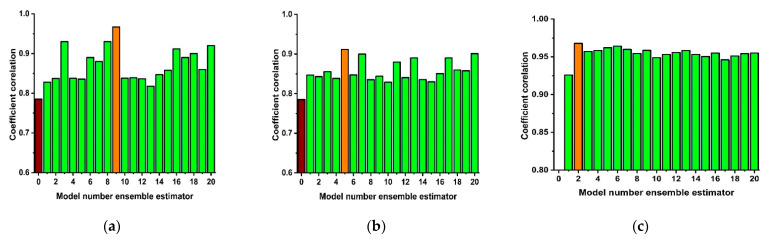
Ensemble models with various number of ensemble estimators; (**a**) SVR-bagging; (**b**) SVR-boosting; (**c**) Random forest.

Similarly, in the boosting technique, the cumulative models are developed, and several components are formed having higher precision than individual models. In the boosting technique, the sub-models are assembled in finals model based on the weighted average of the dependent sub-models. In this research, the SVM regression technique is employed as a base learner along with ensemble algorithms, i.e., bagging, boosting, and RF technique, to predict the compressive strength of LFC. In the current study, the ensemble learners (1 each) with 1, 2, 3, ………, 20 sub-model components were employed to select the optimum range of base learners, and the best construction was chosen based on the coefficient of correlation (R) values. The performance of various ensemble models against different sub-model components is shown in Figure 3. Figure 3a shows the SVR-bagging ensemble, where 9 sub-models develop a strong correlation, and the prominent effect of sub-models on boosting and RF ensemble models is shown in Figure 3b,c. This initial analysis shows an enhancement in the individual learner performance with the incorporation of ensemble learners. The chosen architectures for ensemble learners are described in Table 4.

### 3.3. 10-K Fold Cross-Validation and Statistical Evaluation

The 10-K fold cross-validation algorithms are used to minimize the random sampling of training and hold-out data sets. A reliable variance within the optimal computational time is obtained from the 10 K-fold validation approach [79]. In this study, a statistical 10-K fold approach was applied to evaluate the performance of developed models, which distributes a data set into ten equal subsets. For model development and validation, a unique data subset for training and testing was taken with other data subsets in each of the ten rounds. The algorithm accuracy in 10-validation rounds for ten models is expressed as an average accuracy.

Furthermore, different statistical regression and error tools were used to evaluate and gauge the performance of the developed models and are given in Equations (6)–(8). Different researches suggest that the models having a high value of R^2^ and low values of statistical error are considered accurate and reliable [46,80].

(6) $$R=\frac{\sum_{i=1}^{n}\left(ai-\bar{a}\right)\left(pi-\bar{p}\right)}{\sqrt{\sum_{i=1}^{n}\left(ai-\bar{a}\right)2\sum_{i=1}^{n}\left(pi-\bar{p}\right)2}}$$

(7) $$\mathrm{MAE}=\frac{\sum_{i=1}^{n}\left|ai-pi\right|}{n}$$

(8) $$\mathrm{RMSE}=\sqrt{\frac{\sum_{i=1}^{n}{\left(ai-pi\right)}^{2}}{n}}$$

where *ai = i*th actual value and *pi* = *i*th prediction value. $\bar{a}$ = average of actual output values, $\bar{p}$ = average of the prediction output, and *n* = the total number of data points.

## 4. Model Results and Discussion

### 4.1. Results of Support Vector Machine Regression with Ensemble Learner

**Figure 4:**
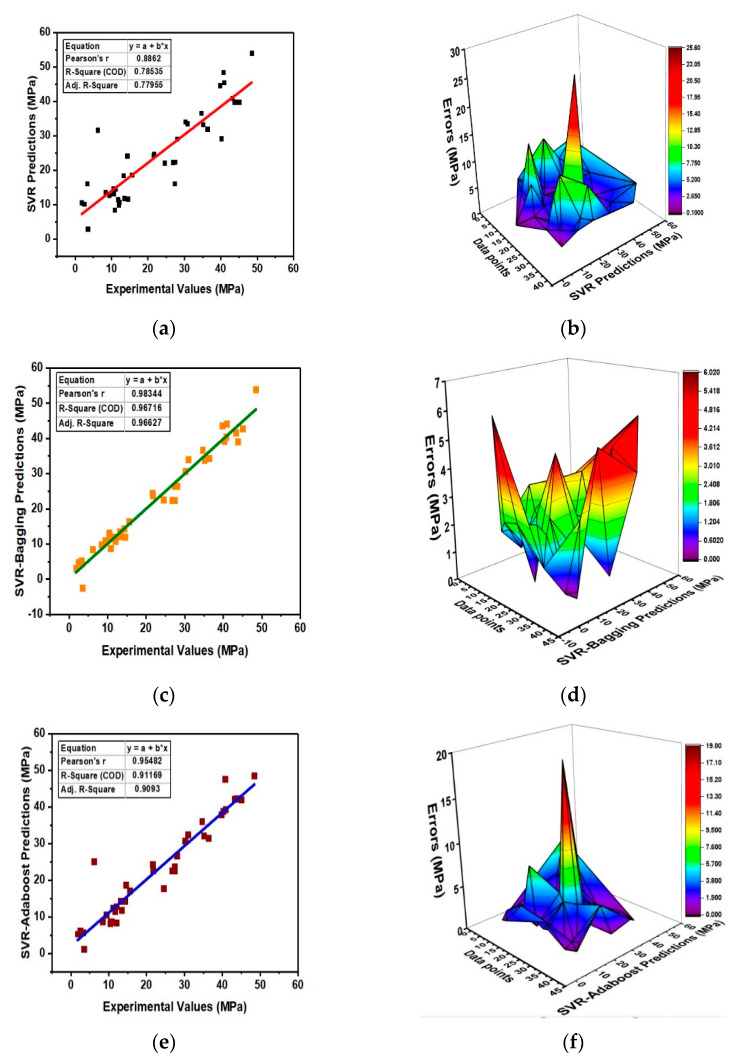
(**a**) SVR relation and (**b**) error distribution; (**c**) SVR-Bagging relation and (**d**) error distribution; (**e**) SVR-Adaboost relation and (**f**) error distribution between experimental and prediction values.

Figure 4 shows the prediction results of SVM regression and the ensemble models along with the prediction error distribution graphs. The individual SVM model yields a correlation of R^2^ = 0.78 and the ensemble model yields R^2^ = 0.96 and R^2^ = 0.91 for bagging and boosting models, respectively, as shown in Figure 4a,c,e. From Figure 4b, the error distribution graph shows an average error of 4.96 MPa for the SVM regression model and that for bagging and boosting, an average error of 2.05 MPa and 2.72 MPa was recorded, respectively, as shown in Figure 4d,f. The result also shows that 80% of the individual SVM model results have error values less than 6 MPa, and that for both bagging and boosting, 92% of the model results have error values less than 5 MPa. It is observed from the results that the ensemble learning models have a strong prediction capability as compared to the individual SVM regression model. Moreover, the robustness of the models is also depicted by statistical analysis. Table 5 represents the statistical evaluation of the models.

### 4.2. Results of Random Forest Regression

**Figure 5:**
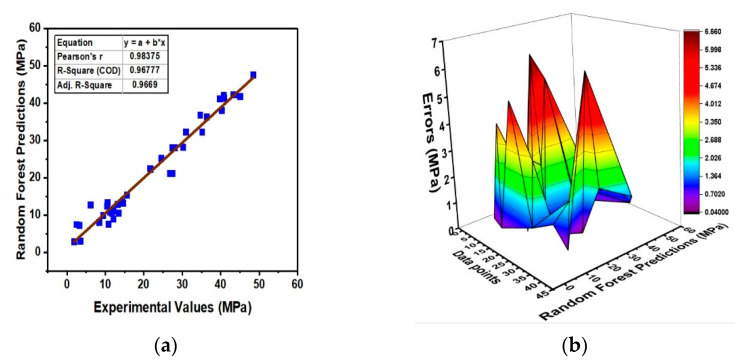
Results of random forest ML approach; (**a**) regression relation between experimental and prediction values; (**b**) Prediction errors distribution.

Random forest is a modified ensemble ML technique that combines the bagging ensemble learner and random feature selection, which is user-friendly and can be employed for the development of reliable prediction models. Better accuracy in the prediction of compressive strength of LFC has been achieved by employing the RF technique and is shown in Figure 5. Figure 5a shows a strong correlation of R^2^ = 0.96 between the experimental and RF prediction values. From Figure 5b, it can be seen that 90% of the data points have error values less than 5 MPa and have a maximum and minimum error value of 6.65 MPa and 0.015 MPa, respectively. An average prediction error value of 1.85 MPa was recorded for the RF regression model. The low values of prediction errors and high value of the coefficient of determinant (R^2^) manifest that the performance of prediction models can be enhanced with the application of ensemble and modified ensemble techniques and better accuracy can be achieved.

The statistical evaluation of the developed ML models is illustrated in Table 5. The individual SVR model performance is enhanced with the application of ensemble techniques and the coefficient of regression R^2^ is increased from 0.81 for SVR to 0.96 for the SVR-bagging model. Similarly, after the application of ensemble learners, the statistical error values also reduced significantly. For example, the MAE value for SVR is recorded as 4.96 MPa, which is reduced to 2.05 MPa for SVR-bagging ensemble learners. The modified ensemble learner (RF) outperforms all the ML techniques used in this research and yields R^2^ = 0.96 along with the least statistical error values of MAE = 1.84 MPa and RMSE = 2.52 MPa, proving to be a more efficient technique with adamant results.

### 4.3. 10-K Fold Cross-Validation and Statistical Evaluation

**Figure 6:**
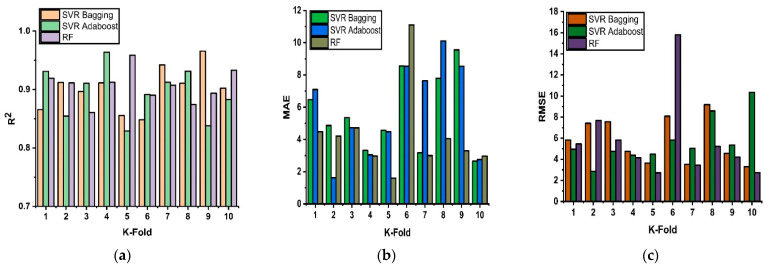
(**a**) Regression results (R^2^) of models with 10 k-fold cross-validations of models; (**b**) MAE Statistical error results of models with 10-K fold cross- validation; (**c**) RMSE Statistical error results of models with 10-k fold cross- validation.

A desired level of accuracy is required for the validity of prediction models. The 10 K-fold cross-validation method is used to ensure the accuracy of the model by shuffling the available data. By using this technique, the bias associated with a random sampling of training data set is minimized. This technique divides the experimental data samples into equal ten subsets and utilizes the nine subsets for developing and shaping the strong learner. Meanwhile, the last subset is utilized to gauge the validity of the developed model. The validation process repeats for ten times, and at the end, the average accuracy is obtained from the ten times repetition. The generalization performance and the reliability of the model are well represented by 10 K-fold cross-validations [79]. The cross-validation tests for individual non-linear, ensemble, and modified ensemble models are represented in Figure 6. The results show that with the application of ensemble techniques, the performance of the model is enhanced from a weak to strong relation along with adamant results. The results of 10 K-fold cross-validations are assessed by using the coefficient of determinant R^2^ (regression tool) along with MAE and RMSE (statistical error tools). In Figure 6a, fluctuation in the value R^2^ is observed for the 10 K-fold validation of different ML techniques, but still, a high level of accuracy is maintained in each fold. For example, the range of R^2^ values for SVR-Bagging, SVR-Boosting, and RF is 0.84–0.96, 0.82–0.96, and 0.86–0.95, respectively. The accuracy of the cross-validation was also assessed in terms of MAE and RMSE and is given in Figure 6b,c, respectively. The average value of MAE for SVR-bagging, SVR-Adaboost, and RF are 5.6 MPa, 5.8 MPa, and 4.2 MPa, respectively, as shown in Figure 6b. Figure 6c shows the RMSE values of 10 K-fold validation and gives an average value of 5.7 MPa, 5.6 MPa, and 5.7 MPa for SVR-bagging, SVR-Adaboost, and RF, respectively. The results of the 10 K-fold cross-validation method reflect the accuracy and reliability of the concerned developed models.

## 5. Conclusions

The different machine learning approaches, individual learner and ensemble learners, are used to predict and estimate the compressive strength of lightweight foamed concrete. The conclusions based on this analysis are given as follow.

(1) The performance of the individual SVR learner has significantly increased with the application of bagging and boosting ensemble learners. The modified ensemble learner (RF) has enhanced the performance of the prediction model by 23% when compared to the individual SVR learner and yields a high correlation of R^2^ = 0.96.
(2) In the 10-fold cross-validation method, all the ensemble learning approaches maintained high accuracy along with the lowest statistical error values of MAE and RMSE.
(3) The statistical evaluation was performed using MAE, RMSE, and R^2^. The modified ensemble learner (RF) approach shows a reduced error of about 62% for both MAE and RMSE as compared to individual SVR learners.
(4) The SVR-bagging reports 58% and 61% lower error values of MAE and RMSE, respectively, as compared to individual SVR learners, and an enhancement of 20% in the robustness of the performance was observed, yielding R^2^ = 0.96.
(5) The SVR-boosting approach records 45% and 38% lower values of MAE and RMSE, respectively, and yields R^2^ = 0.91 with a 17% enhancement in model performance as compared to individual SVR learners.

## Figures and Tables

**Table 1:** Statistics of collected data.

Variable	Unit	Role	Minimum	Maximum	Average	Standard Deviation
Cement content	(kg/m^3^)	Input	292.2	992.8	661.578	174.62
Sand content	(kg/m^3^)	Input	0	1355	699.622	233.629
water/cement	-	Input	0.3	0.84	0.42623	0.10244
Foam volume	(dm^3^/m^3^)	Input	47	690	245.431	121.496
Compressive strength	(MPa)	Output	1.09	48.88	23.9598	13.5282

**Table 2:** Pearson-correlation matrix for mix design parameters.

	Cement	Sand	w/c	Foam	Compressive-Strength
Cement	1				
Sand	0.026	1			
w/c	−0.576	−0.285	1		
Foam	−0.770	−0.485	0.388	1	
Compressive Strength	0.777	0.402	−0.631	−0.748	1

**Table 3:** Summarize machine-learning algorithm by researchers.

Sr. No	Machine Learning Method	Abbreviation	Data Set	Prediction Property	Year	Waste Materials	References
1.	Gene expression programming	GEP	298	Compressive Strength	2021	FA	[29]
2.	Support Vector Machine	SVM	15	Compressive strength	2021	Normal concrete	[49]
3.	Individuals with ensemble modeling	ANN, bagging and boosting	1030	Compressive strength	2021	FA GGBFS	[30]
4.	Data Envelopment Analysis	DEA	114	Compressive strength, Slump test, L-box test, V-funnel test	2021	FA	[50]
5.	Gene expression programming	GEP	160	Post-fire behavior	2020	GGBFS	[51]
6.	Gene expression programming	GEP	351	Compressive Strength	2020	GGBFS	[52]
7.	Multivariate	MV	21	Compressive strength	2020	Crumb rubber with SF	[53]
8.	Support Vector Machine Adaptive-Network-based Fuzzy Inference System	SVM-ANFIS	120	Deflection	2020	RC beam	[54]
9.	Conventional Artificial-Neural Network	C-ANN	220	Compressive Strength	2020	Foamed concrete	[55]
10.	Gene Expression Programming	GEP	357	Compressive strength	2020	Superplasticizers	[56]
11.	Adaptive neuro-fuzzy inference system	ANFIS with ANN	7	Compressive strength	2020	POFA	[57]
12.	Gene expression programming and random forest	GEP and RF	357	Compressive strength	2020	-	[58]
13.	Gene expression programming	GEP	277	Axial capacity	2020	-	[32]
14.	Support vector machine	SVM	-	Compressive strength	2020	FA	[59]
15.	Support vector machine	SVM	115	Slump test, L-box test, V-funnel test, Compressive strength	2020	FA	[60]
16.	Ensemble models	RT, RF, GBRT, ensemble GBRT	126	Unconfined compressive strength	2019	Cemented Paste Backfill	[61]
17.	Artificial Neural-Network	ANN	264	Thermal properties	2019	Silica fume	[62]
18.	Random forest	RF	131	Compressive strength	2019	FA GGBFS SF	[38]
19.	Artificial neuron-network	ANN	205	Compressive strength	2019	FA GGBFS SF RHA	[63]
20.	Intelligent rule-based enhanced multiclass support vector machine and fuzzy rules	IREMSVM-FR with RSM	114	Compressive strength	2019	FA	[64]
21.	Adaptive neuro-fuzzy inference system	ANFIS	55	Compressive strength	2018	-	[65]
22.	Multivariate adaptive regression spline	M5 MARS	114	Compressive strength Slump test L-box test V-funnel test	2018	FA	[66]
23.	Random Kitchen Sink Algorithm	RKSA	40	V-funnel test J-ring test Slump test Compressive strength	2018	FA	[67]
24.	Artificial neuron-network	ANN	69	Compressive strength	2017	FA	[68]
25.	Artificial neuron-network	ANN	114	Compressive strength	2017	FA	[69]
26.	Support Vector Machine Random forest AdaBoost	SVM RF AB	288	Compressive Strength	2017	Blast furnace slag and waste tire rubber powder	[70]
27.	Artificial neuron-network	ANN	169	Compressive strength	2016	FA GGBFS SF RHA	[71]
28.	Biogeographical-based programming	BBP	413	Elastic modulus	2016	SF FA SLAG	[72]
29.	Artificial Neural Network Multi Linear Regression	ANN and MLR	1288	Compressive strength	2015	Clinker mortar	[73]
30.	Gene expression programming	GEP	168	Tensile Strength	2012	Normal concrete	[74]
31.	Artificial neuron-network	ANN	80	Compressive strength	2011	FA	[35]
32.	Artificial neuron-network	ANN	300	Compressive strength	2009	FA	[75]

**Table 4:** Analysis method for optimum sub-models.

Approach	Ensemble Method	ML Technique	Ensemble Models	Optimum Estimator	R-Value
Individual	-	Support vector regression	-	-	0.88
Ensemble learner	Bagging	SVR-Bagging	(1, 2, 3, …., 20)	9	0.98
Ensemble learner	Boosting	SVR-Boosting	(1, 2, 3, …., 20)	5	0.95
Modified ensemble	-	Random Forest	(1, 2, 3, …., 20)	2	0.98

**Table 5:** Statistical evaluation of different ML modeling approaches.

ML Technique	Approach	MAE (MPa)	RMSE (MPa)	R^2^
Support vector regression	Individual	4.96	6.68	0.78
SVR-Bagging	Ensemble learner	2.05	2.54	0.96
SVR-Boosting	Ensemble learner	2.72	4.12	0.91
Random Forest	Modified ensemble learner	1.84	2.52	0.96

## Data Availability

The data will be available on request.

## Notations

LFC	lightweight foamed concrete
SVM	support vector machine
ML	machine learning
AI	artificial intelligence
RF	random forest
R^2^	coefficient of determinant
MAE	mean absolute error
GHG	greenhouse gases
CO_2_	carbon dioxide
DT	decision tree
DL	deep learning
ANN	artificial neural network
r	Pearson correlation
SVR	support vector regression
CS	compressive strength
RMSE	root mean square error

## References

[1] Akbar A., Farooq F., Shafique M., Aslam F., Alyousef R., Alabduljabbar H. (2021). Sugarcane bagasse ash-based engineered geopolymer mortar incorporating propylene fibers. J. Build. Eng..

[2] Farooq F., Jin X., Javed M.F., Akbar A., Shah M.I., Aslam F., Alyousef R. (2021). Geopolymer concrete as sustainable material: A state of the art review. Constr. Build. Mater..

[3] Richard A.O., Ramli M. (2013). Experimental production of sustainable lightweight foamed concrete. Br. J. Appl. Sci. Technol..

[4] Shah S.N., Mo K.H., Yap S.P., Yang J., Ling T.C. (2021). Lightweight foamed concrete as a promising avenue for incorporating waste materials: A review. Resour. Conserv. Recycl..

[5] Mehta K.P. (2001). Reducing the environmental impact of concrete. Concr. Int..

[6] Zabalza Bribián I., Valero Capilla A., Aranda Usón A. (2011). Life cycle assessment of building materials: Comparative analysis of energy and environmental impacts and evaluation of the eco-efficiency improvement potential. Build. Environ..

[7] Narayanan N., Ramamurthy K. (2000). Structure and properties of aerated concrete: A review. Cem. Concr. Compos..

[8] Kearsley E.P. (1999). Just foamed concrete—An overview. Proceedings of the Creating with Concrete: Proceedings International Conference (and Seminars), University of Dundee.

[9] Amran Y.H.M., Farzadnia N., Ali A.A.A. (2015). Properties and applications of foamed concrete; a review. Constr. Build. Mater..

[10] Raj A., Sathyan D., Mini K.M. (2019). Physical and functional characteristics of foam concrete: A review. Constr. Build. Mater..

[11] Jhatial A.A., Goh W.I., Mohamad N., Hong L.W., Lakhiar M.T., Samad A.A.A., Abdullah R. (2018). The mechanical properties of foamed concrete with polypropylene fibres. Int. J. Eng. Technol..

[12] Rahman N.A., Jaini Z.M., Rahim N.A.A., Razak S.A.A. (2015). An experimental study on the fracture energy of foamed concrete using v-notched beams. InCIEC 2014.

[13] Tikalsky P.J., Pospisil J., MacDonald W. (2004). A method for assessment of the freeze-thaw resistance of preformed foam cellular concrete. Cem. Concr. Res..

[14] Mohamad N., Muhammad H.M. (2011). Testing of precast lightweight foamed concrete sandwich panel with single and double symmetrical shear truss connectors under eccentric loading. Advanced Materials Research.

[15] Kunhanandan Nambiar E.K., Ramamurthy K. (2008). Fresh state characteristics of foam concrete. J. Mater. Civ. Eng..

[16] Jones M.R., McCarthy A. (2005). Preliminary views on the potential of foamed concrete as a structural material. Mag. Concr. Res..

[17] Jones M.R., McCarthy A. (2006). Heat of hydration in foamed concrete: Effect of mix constituents and plastic density. Cem. Concr. Res..

[18] Hamidah M.S., Azmi I., Ruslan M.R.A., Kartini K., Fadhil N.M. (2005). Optimisation of foamed concrete mix of different sand-cement ratio and curing conditions. Use of Foamed Concrete in Construction: Proceedings of the International Conference Held at the University of Dundee, Scotland, UK, 5 July 2005.

[19] Kearsley E.P., Wainwright P.J. (2001). The effect of high fly ash content on the compressive strength of foamed concrete. Cem. Concr. Res..

[20] Valore R.C. (1954). Cellular concretes part 2 physical properties. J. Proc..

[21] Dhir R.K., Newlands M.D., McCarthy A. (2009). Use of Foamed Concrete in Construction.

[22] Nehdi M., Djebbar Y., Khan A. (2001). Neural network model for preformed-foam cellular concrete. Mater. J..

[23] Neville A.M. (1995). Properties of Concrete.

[24] Khaloo A.R., Dehestani M., Rahmatabadi P. (2008). Mechanical properties of concrete containing a high volume of tire-rubber particles. Waste Manag..

[25] Li M., Hao H., Shi Y., Hao Y. (2018). Specimen shape and size effects on the concrete compressive strength under static and dynamic tests. Constr. Build. Mater..

[26] Feng D.-C., Liu Z.-T., Wang X.-D., Chen Y., Chang J.-Q., Wei D.-F., Jiang Z.-M. (2020). Machine learning-based compressive strength prediction for concrete: An adaptive boosting approach. Constr. Build. Mater..

[27] Farooq F., Czarnecki S., Niewiadomski P., Aslam F., Alabduljabbar H., Ostrowski K.A., Śliwa-Wieczorek K., Nowobilski T., Malazdrewicz S. (2021). A comparative study for the prediction of the compressive strength of self-compacting concrete modified with fly ash. Materials.

[28] Song H., Ahmad A., Farooq F., Ostrowski K.A., Maślak M., Czarnecki S., Aslam F. (2021). Predicting the compressive strength of concrete with fly ash admixture using machine learning algorithms. Constr. Build. Mater..

[29] Khan M.A., Memon S.A., Farooq F., Javed M.F., Aslam F., Alyousef R. (2021). Compressive strength of fly-ash-based geopolymer concrete by gene expression programming and random forest. Adv. Civ. Eng..

[30] Farooq F., Ahmed W., Akbar A., Aslam F., Alyousef R. (2021). Predictive modeling for sustainable high-performance concrete from industrial wastes: A comparison and optimization of models using ensemble learners. J. Clean. Prod..

[31] Ahmad A., Farooq F., Ostrowski K.A., Śliwa-Wieczorek K., Czarnecki S. (2021). Application of novel machine learning techniques for predicting the surface chloride concentration in concrete containing waste material. Materials.

[32] Javed M.F., Farooq F., Memon S.A., Akbar A., Khan M.A., Aslam F., Alyousef R., Alabduljabbar H., Rehman S.K.U., Ur Rehman S.K. (2020). New prediction model for the ultimate axial capacity of concrete-filled steel tubes: An evolutionary approach. Crystals.

[33] Ahmad A., Ahmad W., Aslam F., Joyklad P. (2022). Compressive strength prediction of fly ash-based geopolymer concrete via advanced machine learning techniques. Case Stud. Constr. Mater..

[34] Li Z., Yim S.H.-L., Ho K.-F. (2020). High temporal resolution prediction of street-level PM2. 5 and NOx concentrations using machine learning approach. J. Clean. Prod..

[35] Siddique R., Aggarwal P., Aggarwal Y. (2011). Prediction of compressive strength of self-compacting concrete containing bottom ash using artificial neural networks. Adv. Eng. Softw..

[36] Dantas A.T.A., Batista Leite M., De Jesus Nagahama K. (2013). Prediction of compressive strength of concrete containing construction and demolition waste using artificial neural networks. Constr. Build. Mater..

[37] Chou J.-S., Tsai C.-F., Pham A.-D., Lu Y.-H. (2014). Machine learning in concrete strength simulations: Multi-nation data analytics. Constr. Build. Mater..

[38] Zhang J., Ma G., Huang Y., Sun J., Aslani F., Nener B. (2019). Modelling uniaxial compressive strength of lightweight self-compacting concrete using random forest regression. Constr. Build. Mater..

[39] Ahani I.K., Salari M., Shadman A. (2020). An ensemble multi-step-ahead forecasting system for fine particulate matter in urban areas. J. Clean. Prod..

[40] Ahmad M.R., Chen B., Yu J. (2019). A comprehensive study of basalt fiber reinforced magnesium phosphate cement incorporating ultrafine fly ash. Compos. Part B Eng..

[41] Abd A.M., Abd S.M. (2017). Modelling the strength of lightweight foamed concrete using support vector machine (SVM). Case Stud. Constr. Mater..

[42] Asadzadeh S., Khoshbayan S. (2018). Multi-objective optimization of influential factors on production process of foamed concrete using box-behnken approach. Constr. Build. Mater..

[43] Abdulrahman Hilal A., Thom N., Dawson A. (2015). The use of additives to enhance properties of pre-formed foamed concrete. Int. J. Eng. Technol..

[44] Mounanga P., Gbongbon W., Poullain P., Turcry P. (2008). Proportioning and characterization of lightweight concrete mixtures made with rigid polyurethane foam wastes. Cem. Concr. Compos..

[45] Pan Z., Hiromi F., Wee T. (2007). Preparation of high performance foamed concrete from cement, sand and mineral admixtures. J. Wuhan Univ. Technol. Sci. Ed..

[46] Gandomi A.H., Roke D.A. (2015). Assessment of artificial neural network and genetic programming as predictive tools. Adv. Eng. Softw..

[47] Azim I., Yang J., Javed M.F., Iqbal M.F., Mahmood Z., Wang F., Liu Q.F. (2020). Prediction model for compressive arch action capacity of RC frame structures under column removal scenario using gene expression programming. Structures.

[48] DeGhett V.J. (2014). Effective use of pearson’s product-moment correlation coefficient: An additional point. Anim. Behav..

[49] Lv Z., Jiang A., Jin J., Lv X. (2021). Multifractal analysis and compressive strength prediction for concrete through acoustic emission parameters. Adv. Civ. Eng..

[50] Balf F.R., Kordkheili H.M., Kordkheili A.M. (2021). A new method for predicting the ingredients of self-compacting concrete (SCC) including fly ash (FA) using data envelopment analysis (DEA). Arab. J. Sci. Eng..

[51] Fakhrian S., Behbahani H., Mashhadi S. (2020). Predicting post-fire behavior of green geopolymer mortar containing recycled concrete aggregate via GEP approach. J. Soft Comput. Civ. Eng..

[52] Shahmansouri A.A., Bengar H.A., Ghanbari S. (2020). Compressive strength prediction of eco-efficient GGBS-based geopolymer concrete using GEP method. J. Build. Eng..

[53] Bušić R., Benšić M., Miličević I., Strukar K. (2020). Prediction models for the mechanical properties of self-compacting concrete with recycled rubber and silica fume. Materials.

[54] Bai C., Nguyen H., Asteris P.G., Nguyen-Thoi T., Zhou J. (2020). A refreshing view of soft computing models for predicting the deflection of reinforced concrete beams. Appl. Soft Comput. J..

[55] Dao D.V., Ly H.-B.B., Vu H.-L.T.L.T., Le T.-T.T., Pham B.T. (2020). Investigation and optimization of the C-ANN structure in predicting the compressive strength of foamed concrete. Materials.

[56] Aslam F., Farooq F., Amin M.N., Khan K., Waheed A., Akbar A., Javed M.F., Alyousef R., Alabdulijabbar H. (2020). Applications of gene expression programming for estimating compressive strength of high-strength concrete. Adv. Civ. Eng..

[57] Al-Mughanam T., Aldhyani T.H.H., Alsubari B., Al-Yaari M. (2020). Modeling of compressive strength of sustainable self-compacting concrete incorporating treated palm oil fuel ash using artificial neural network. Sustainability.

[58] Farooq F., Amin M.N., Khan K., Sadiq M.R., Javed M.F., Aslam F., Alyousef R. (2020). A comparative study of random forest and genetic engineering programming for the prediction of compressive strength of high strength concrete (HSC). Appl. Sci..

[59] Azimi-Pour M., Eskandari-Naddaf H., Pakzad A. (2020). Linear and non-linear SVM prediction for fresh properties and compressive strength of high volume fly ash self-compacting concrete. Constr. Build. Mater..

[60] Saha P., Debnath P., Thomas P. (2020). Prediction of fresh and hardened properties of self-compacting concrete using support vector regression approach. Neural Comput. Appl..

[61] Lu X., Zhou W., Ding X., Shi X., Luan B., Li M. (2019). Ensemble learning regression for estimating unconfined compressive strength of cemented paste backfill. IEEE Access.

[62] Fidan S., Oktay H., Polat S., Ozturk S. (2019). An artificial neural network model to predict the thermal properties of concrete using different neurons and activation functions. Adv. Mater. Sci. Eng..

[63] Asteris P.G., Kolovos K.G. (2019). Self-compacting concrete strength prediction using surrogate models. Neural Comput. Appl..

[64] Selvaraj S., Sivaraman S. (2019). Prediction model for optimized self-compacting concrete with fly ash using response surface method based on fuzzy classification. Neural Comput. Appl..

[65] Vakhshouri B., Nejadi S. (2018). Prediction of compressive strength of self-compacting concrete by ANFIS models. Neurocomputing.

[66] Kaveh A., Bakhshpoori T., Hamze-Ziabari S.M. (2018). M5′ and mars based prediction models for properties of selfcompacting concrete containing fly ash. Period. Polytech. Civ. Eng..

[67] Sathyan D., Anand K.B., Prakash A.J., Premjith B. (2018). Modeling the fresh and hardened stage properties of self-compacting concrete using random kitchen sink algorithm. Int. J. Concr. Struct. Mater..

[68] Abu Yaman M., Abd Elaty M., Taman M. (2017). Predicting the ingredients of self compacting concrete using artificial neural network. Alex. Eng. J..

[69] Belalia Douma O., Boukhatem B., Ghrici M., Tagnit-Hamou A. (2017). Prediction of properties of self-compacting concrete containing fly ash using artificial neural network. Neural Comput. Appl..

[70] Ozcan G., Kocak Y., Gulbandilar E. (2017). Estimation of compressive strength of BFS and WTRP blended cement mortars with machine learning models. Comput. Concr..

[71] Asteris P.G., Kolovos K.G., Douvika M.G., Roinos K. (2016). Prediction of self-compacting concrete strength using artificial neural networks. Eur. J. Environ. Civ. Eng..

[72] Golafshani E.M., Ashour A. (2016). Prediction of self-compacting concrete elastic modulus using two symbolic regression techniques. Autom. Constr..

[73] Beycioglu A., Emiroglu M., Kocak Y., Subaşi S. (2015). Analyzing the compressive strength of clinker mortars using approximate reasoning approaches—ANN vs. MLR. Comput. Concr..

[74] Severcan M.H. (2012). Prediction of splitting tensile strength from the compressive strength of concrete using GEP. Neural Comput. Appl..

[75] Prasad B.K.R., Eskandari H., Reddy B.V.V. (2009). Prediction of compressive strength of SCC and HPC with high volume fly ash using ANN. Constr. Build. Mater..

[76] Han Q., Gui C., Xu J., Lacidogna G. (2019). A generalized method to predict the compressive strength of high-performance concrete by improved random forest algorithm. Constr. Build. Mater..

[77] Han J., Pei J., Kamber M. (2011). Data Mining: Concepts and Techniques.

[78] Zhang J., Huang Y., Aslani F., Ma G., Nener B. (2020). A hybrid intelligent system for designing optimal proportions of recycled aggregate concrete. J. Clean. Prod..

[79] Kohavi R. (1995). A study of cross-validation and bootstrap for accuracy estimation and model selection. Int. Jt. Conf. Artif. Intell..

[80] Babanajad S.K., Gandomi A.H., Alavi A.H. (2017). New prediction models for concrete ultimate strength under true-triaxial stress states: An evolutionary approach. Adv. Eng. Softw..

